# Evidence of the presence of *Borrelia burgdorferi* in dogs and associated ticks in Egypt

**DOI:** 10.1186/s12917-020-02733-5

**Published:** 2021-01-25

**Authors:** R. Elhelw, M. Elhariri, D. Hamza, M. Abuowarda, E. Ismael, H. Farag

**Affiliations:** 1 Department of Microbiology, Faculty of Veterinary Medicine, Cairo University, Giza, Egypt; 2 Department of Zoonoses, Faculty of Veterinary Medicine, Cairo University, Giza, Egypt; 3 Department of Parasitology, Faculty of Veterinary Medicine, Cairo University, Giza, Egypt; 4 Department of Veterinary Hygiene and Management, Cairo University, Giza, Egypt; 5 Department of Infectious Diseases, Faculty of Veterinary Medicine, Cairo University, Giza, Egypt

**Keywords:** *Borrelia burgdorferi*, Dogs, Camels, Bb.*16S rRNA*, Gene ticks, *R. sanguineus*, ITS2 region

## Abstract

**Background:**

*Borrelia burgdorferi* is the spirochete that causes Lyme Borreliosis (LB), which is a zoonotic tick-borne disease of humans and domestic animals. Hard ticks are obligate haematophagous ectoparasites that serve as vectors of *Borrelia burgdorferi*. Studies on the presence of Lyme borreliosis in Egyptian animals and associated ticks are scarce.

**Methods:**

This study was conducted to detect *B. burgdorferi* in different tick vectors and animal hosts. Three hundred animals (dogs=100, cattle=100, and camels=100) were inspected for tick infestation. Blood samples from 160 tick-infested animals and their associated ticks (*n*=1025) were collected and examined for the infection with *B. burgdorferi* by polymerase chain reaction (PCR) and sequencing of the *16S rRNA* gene. The identified tick species were characterized molecularly by PCR and sequencing of the ITS2 region.

**Results:**

The overall tick infestation rate among examined animals was 78.33% (235/300). The rate of infestation was significantly higher in camels (90%), followed by cattle (76%) and dogs (69%); (*P* = 0.001). *Rhipicephalus sanguineus*, *Rhipicephalus (Boophilus) annulatus*, and both *Hyalomma dromedarii* and *Amblyomma variegatum,* were morphologically identified from infested dogs, cattle, and camels; respectively. Molecular characterization of ticks using the ITS2 region confirmed the morphological identification, as well as displayed high similarities of *R. sanguineus, H. dromedarii,* and *A. Variegatu* with ticks identified in Egypt and various continents worldwide. Just one dog (1.67%) and its associated tick pool of *R. sanguineus* were positive for *B. burgdorferi* infection. The *16S rRNA* gene sequence for *B. burgdorferi* in dog and *R. sanguineus* tick pool showed a 100% homology.

**Conclusion:**

Analyzed data revealed a relatively low rate of *B. burgdorferi* infection, but a significantly high prevalence of tick infestation among domesticated animals in Egypt, which possesses a potential animal and public health risk. Additionally, molecular characterization of ticks using the ITS2 region was a reliable tool to discriminate species of ticks and confirmed the morphological identification.

## Background

Lyme disease (LD) or the tick-borne relapsing fever (TBRF) is an emerging tick-borne multi-systemic zoonotic bacterial disease that has a worldwide distribution, caused by spirochetes of the *Borrelia burgdorferi* group and transmitted by ticks of the *Ixodes ricinus* complex [1]. The bacterium is horizontally transmitted between ticks and within reservoir hosts of wild animals, mostly small mammals, and humans which are considered an incidental host [2]. Clinical signs differ according to the species of *B. burgdorferi* complex prevailing in the area, but most human patients show erythema migrans accompanied by flu-like symptoms [2, 3]. Companion animals, particularly dogs, act as sentinels for Lyme disease [4]. Only 5–10% of infected dogs show clinical signs. Therefore, there is a significant underestimation of the Lyme disease prevalence in dogs, which represents a risk for disease spreading [5]. A previous survey on domestic animals detected borrelial DNA only in the adult ticks, mostly infesting sheep and cattle [6].

Ticks (subclass: Acari; order: Parasitiformes; suborder: Ixodida) are obligate haematophagous ectoparasites of wild and domestic animals and humans. Ticks are considered the most important vectors of disease-causing pathogens within the phylum Arthropoda, being comparable only to mosquitoes (family Culicidae) [7, 8]. As the incidence of tick-borne diseases propagates and expands geographically, it is becoming increasingly significant to distinguish tick species, to enhance tick and tick-borne disease control [9]. The brown tick *Rhipicephalus sanguineus* of dogs [10], the blue tick *Rhipicephalus Boophilus* of cattle [11], *Hyalomma dromedarii* of camels [12], and the tropical bont tick (TBT) *Amblyomma variegatum* of ruminants [13, 14], are of significant veterinary and medical importance due to their vector competence for several pathogens [15].

Despite the existence of various hard tick species in Egypt, limited data on the borrelial infection of these ticks is available. Here, we studied the infection of the hard ticks in Egypt with *B. burgdorferi*. Additionally, we investigated the prevalence and phylogeny of *Borrelia* in the tick-infested dogs, cattle, and camels during the year 2017.

## Results

### Prevalence of tick infestation in the studied animals

*Rhipicephalus sanguineus* was detected in 69% of sampled dogs, *Rhipicephalus (Boophilus) annulatus* was detected in 76% of sampled cattle, and both *Hyalomma dromedarii* and *Amblyomma variegatum* were detected in 90% of examined camels. The relation between animal species and the tick infestation rate was significant, *χ*^*2*^ (2, *N* = 300) = 13.47, *P* = 0.001. Camels were more likely than dogs and cattle to be infested with ticks (Table 1). Camels showed a significantly higher infestation rate with *Hyalomma dromedarii* (93.33%) compared to *Amblyomma variegatum* (6.67%), *χ*^*2*^ (1, *N* = 90) = 135.2, *P* < 0.0001.

**Table 1:** Identified tick species and infestation rate among sampled animals

Animals	No. of inspected animals	Tick species	No. (%) of tick infested animals
**Dog**	100	*Rhipicephalus sanguineus*	69 (69%) ^b^
**Cattle**	100	*Rhipicephalus (Boophilus) annulatus*	76 (76%) ^b^
**Camel**	100		90 (90%) ^a^
		*Hyalomma dromedarii*	*84/90 (93.33%)*
		*Amblyomma variegatum*	*6/90 (6.67%)*
**Total**	300		235 (78.33%)

^a,b^ Different superscripts indicate significant difference at *P* < 0.05

### Species identification and phylogenetic analysis of ticks

**Fig. 1:**
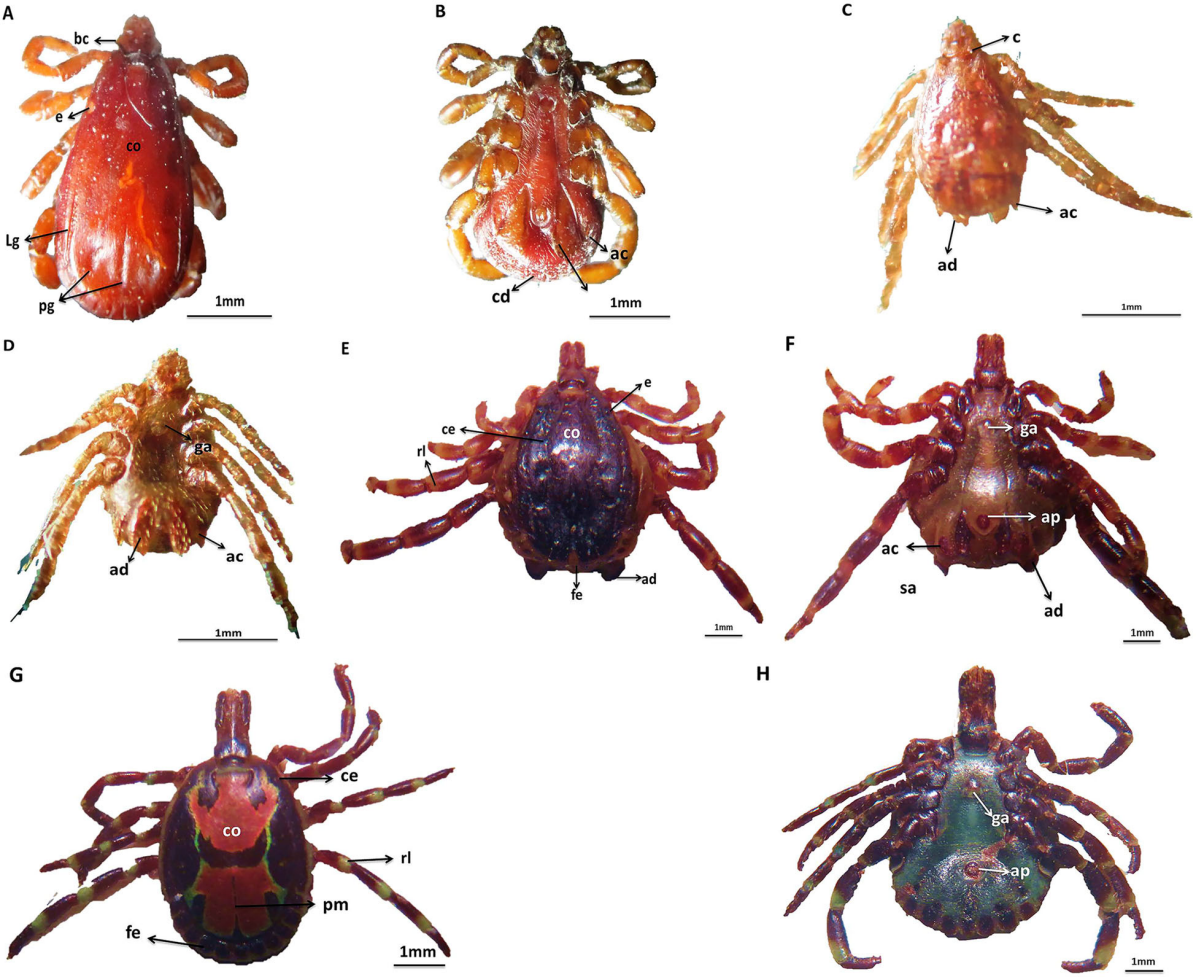
*R. sanguineus* male (**a**&**b**), dorsal view (**a**) showed eyes are slightly convex (e), Basis capital has lateral sharp angles (bc), pale conscutum(co), distinctly lateral grooves (Lg), deep distinct posterior grooves (pg); ventral view (**b**) showed large accessory adenal plate (ac), narrow adenal plate (ad), caudal appendage (cd).*R.(Boophilus) annulatus* male (**c**&**d**), dorsal view (**c**) showed distinctly cornua (c), accessory adanal plates (ac), adanal plates (ad),; ventral view (**d**) showed accessory adanal plates (ac), adanal plates (ad), genital aperture (ga). *H. dromedarii* male(**e**&**f**), dorsal view (**e**) showed conscutum is dark colour (co), eyes(e), rings of leg segments are pale(rl), cervical fields are depressed (ce), central festoons are pale (fe), subanal plates(sa); ventral view (**f**) showed, accessory adanal plates (ac), round end of adanal plates (ad), subanal plates is outside the alignment of adanal plates (sa), genital aperture (ga), anus aperture (ap). *A. Variegatum* male (**g**&**h**), dorsal view (**g**) showed posterior median stripe is narrow (pm), convex eyes are distinct(ce) pale ring of leg segments are distinct (rl), orange to pink enamel colour of conscutum (co), festoons colour are non-enamelled (fe); ventral view(**h**) showed genital aperture (ga), anus aperture(ap)

Ninety percent of examined camels from Cairo (Bassatin abattoir) and Matrouh governorates were infested with two species; *H. dromedarii* (93.3%) and *A. variegatum* (6.6%). Moreover, 76% of examined cattle found in Giza and Elbehira governorates, were infested with *R. (Boophilus) annulatus*. Additionally, 69% of dogs (stray dogs and other breeds) found in Cairo and Giza governorates infested with *R. sanguineus* (Table 1, Fig. 1).

**Fig. 2:**
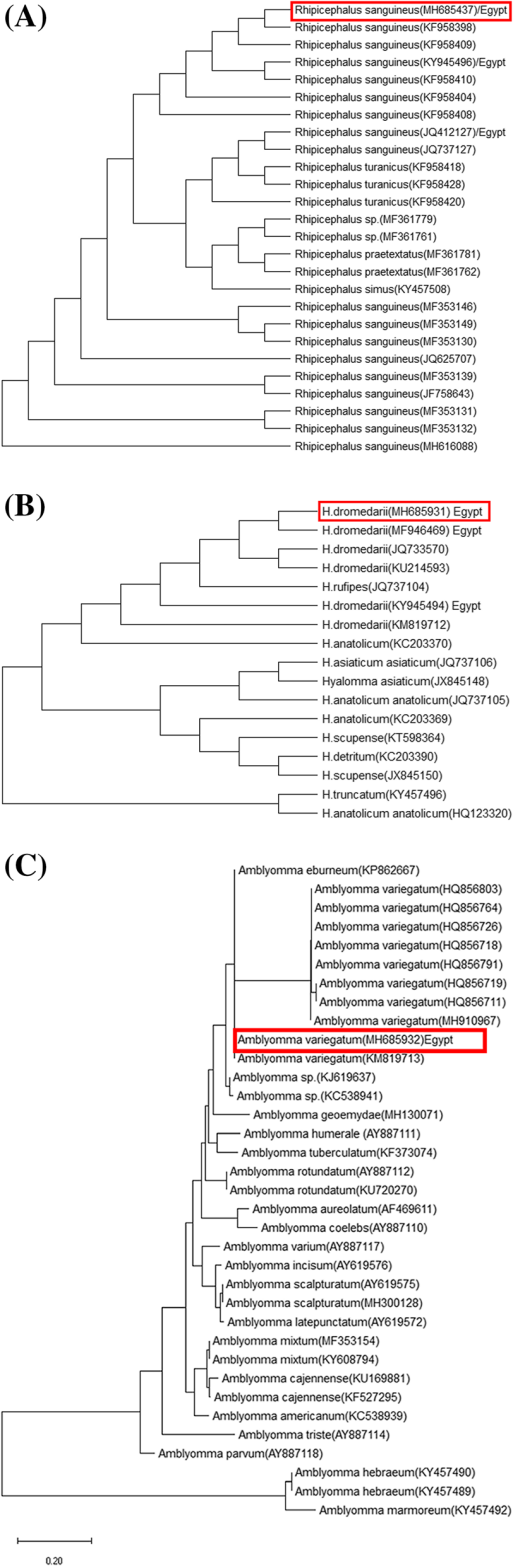
Phylogenetic relationships based on the ITS2 region sequences of ticks. The trees were constructed and analyzed by neighbour-joining (**a**) *R. sanguineus* (**b**) *H. dromedarii* (**c**) *A. Variegatum*

Phylogenetic analysis, based on the ITS2 region sequences of ticks, revealed relatedness of Egyptian ticks *R. sanguineus*, *H. dromedarii*, and *A. Variegatu* with other tick isolated from different continent all over the world, as shown in (Fig. 2).

### Detection and phylogenetic analysis of *B. burgdorferi*

**Fig. 3:**
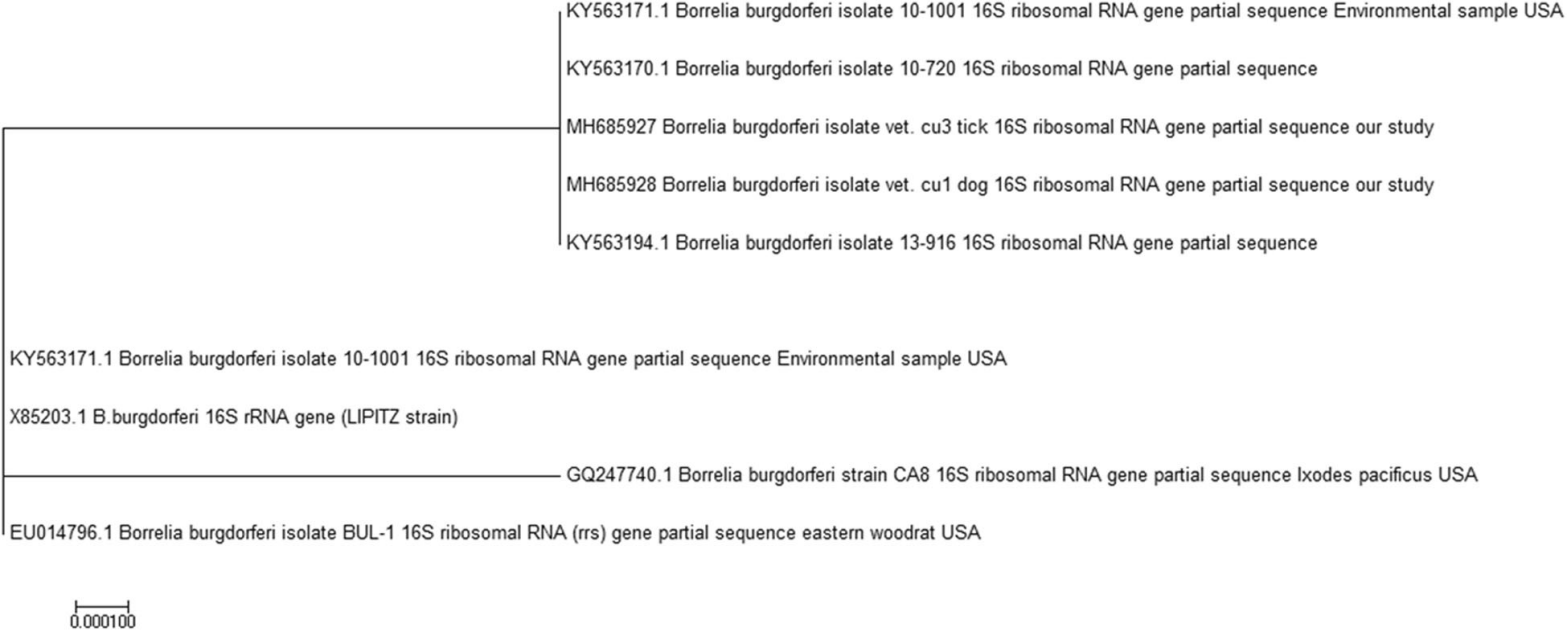
Phylogenetic relationships based on the 16S rRNA gene sequences of *B. burgdorferi*. The trees were constructed and analyzed by neighbour-joining

*Borrelia burgdorferi* was detected in a *Rhipicephalus sanguineus* tick collected from a dog that was also infected (Tables 1 and 2). Based on the *16S rRNA* gene of *B. burgdorferi* collected from dog blood and ticks, a hundred percent of identity were reported between *B. burgdorferi* isolated from the blood of dogs (MH685928) and brown dog ticks (MH685927) in Egypt, as well as *B. burgdorferi* isolated from *Ixodes pacificus* in the USA (KY563172) (Fig. 3).

**Table 2:** *B. burgdorferi* blood and tick’s PCR test results

	Blood samples		Tick samples	
	Total examined	Number (%) positive	Pools examined	Number (%) positive
**Dogs**	60	1 (1.67%) ^a^	60	1 (1.67%) ^b^
**Cattle**	50	0	50	0
**Camels**	50	0	50	0
**Total**	160	1 (0.63%)	160	1 (0.63%)

^a^*B. burgdorferi* isolated from the blood of dogs (Accession no.: MH685928)

^b^*B. burgdorferi* isolated from the brown dog ticks *Rhipicephalus sanguineus* (Accession no.: MH685927)

### Genospecies identification of ticks

## Discussion

Studies on *Borrelia burgdorferi* (Lyme disease) in Egypt are limited, especially in animals and tick vectors. Besides, the recent large-scale movements of humans and animals, as well as the increasing geographical distribution of several tick species, contributed to the growing global threat of tick-borne disease (TBD) [8, 15]. Therefore, in the current study we moleculary identified the hard tick population infesting the examined animals, as well as, investigated the prevalence and phylogeny of *B. burgdorferi* in dogs, cattle, camels, and associated ticks.

In this study, the overall rate of tick infestation in dogs, cattle, and camels, was 78.33% (160/300). The same infestation rate, 78.3% (369/471) in livestock animals, was reported by [4] in Pakistan. Morphological identification of collected ticks indicated that dogs (69%) were infested with *Rhipicephalus sanguineus*, cattle (76%) were infested with *Rhipicephalus (Boophilus) annulatus*, while camels (90%) were infested with either *Hyalomma dromedarii* (93.33%) or *Amblyomma variegatum* (6.67%). *Rhipicephalus sanguineus* (brown dog tick) is the most widespread tick in the world, infesting dogs living in both urban and rural areas [15]. The prevalence of *R. sanguineus* agreed with [16] who reported a 67.5% *R. sanguineus* infestation rate in dogs in Egypt. In southeast Brazil, [17] recorded that 89.7% of dogs were infested with *R. sanguineus*, while in the Philippines, [18] recorded 2.60% prevalence of *R. sanguineus* infestation in dogs. In camels, *Hyalomma dromedarii* was the most abundant tick species, which agrees with the previous study conducted by [19].

Although the morphological identities of identified ticks were similar to the taxonomic key of Ixodidae ticks set by [20], further molecular identification of ticks was carried out through the examination of the ITS2 region. The choice of the ITS2 region was based on the previous studies [21, 22],which indicated that this DNA marker is reliable in discriminating species of ticks. The sequence of the ITS2 region of our isolates showed varying degrees of similarities with local and international hard tick species. The dog tick, *Rhipicephalus sanguineus* (MH685437) showed the highest similarity (99.7%) with *R. sanguineus* obtained from Alexandria (KY945496) and the neighboring countries; as Israel (KF958410, KF958409, KF958404, KF958398). However, it showed a 19.3% homology with *R. sanguineus* (MH616088) collected from Brazil. This difference in similarity agreed with [23–25] who classified *R. sanguineus* group into tropical and temperate strains. *Rhipicephalus annulatus* (MH685437) of cattle, showed a 100% homology with Egyptian strains (MF946470, KY945495) collected from Al Buhayra Governorate, as well as a Romanian strain (KC503267). However, the *Rhipicephalus annulatus* (MH685437) showed 81.6% homology with *R. annulatus* (JQ412126) collected from Fayoum governorate in Egypt. Those intra-species and inter-species similarities could be due to the agreements in the area of sample collection from northern Egypt as well as the same infested cattle population. Genetic identity of the Egyptian *Hyalomma dromederii* (MH685931) collected in this study from camels (*Camelus dromedaries*), proved a high sequence homology of 100% similarity with previous *H. dromederii* isolates from Matrouh in Egypt, with accession no. MF946469, KY945494. On the other hand, the phylogenetic analysis of *A. variegatum* in this study (MH685932) was a pioneer one, as no earlier reports on similar molecular taxonomy or evolutionary analyses on *A. variegatum* was recorded in Egypt. *Amblyomma variegatum* in this study (MH685932) showed a 97.7% homogeneity with *A. variegatum* collected from cattle in France (MH910967) [26] and 99.9% with one collected from wild herbivores in Kenya (KM819713).

The detection of borrelial DNA in infested animals and associated ticks revealed that one dog (1.67%) and its associated pool of adult *Rhipicephalus sanguineus* ticks (1.67%) were infected with *B. burgdorferi*. The low tick infection rate with *B. burgdorferi* reported in our study disagrees with that recorded in Fayoum and Beni-Suif in Egypt, by [27] who reported 7/12 (58.33%) infected pools of *R. sanguineus*. This difference could be assigned to different geographical locations; as Cairo and Giza are more urbanized than Fayoum and Beni-Suif which are mostly rural governorates. The prevalence of *B. burgdorferi* in the examined dogs in Italy was 1.47%. In the USA, infection rates with *B. burgdorferi* recorded 1.2, 4.0, and 6.7% in dogs from three different regions [28, 29]. The most recent investigations about the prevalence of *B. burgdorferi* epidemic in the European canine population exposed different rates, as the highest incidence observed in Poland 40.2% [30, 31] and the lowest was in Portugal 0.2–0.5% [32] In a previous study [6] in Iran, researchers could detect borrelial DNA only in the adult ticks. They attributed the lack of *Borrelia* infection in nymphs to limited nymph samples as they were unable to catch the immature specimen.

In our study, the identity of *B. burgdorferi* that was isolated from the dog’s blood (MH685928) and the associated brown tick pool of *R. sanguineus* (MH685927) in Egypt, as well as *B. burgdorferi* isolated from *Ixodes pacificus* in the USA (KY563172), were 100% in similarity. From the veterinary and public health points of view, scientists should be aware that ticks are active in broad climatic conditions, even greater than those expected. The current level of knowledge about LB risk and the risk related to tick bites is quite limited and underestimated.

## Conclusion

In Egypt, the domestic animal population is markedly infested with the hard ticks, which threatens the animal and public health with potential tick-borne pathogens. Our data revealed that the camel tick *H. dromedarii* is the most prevalent tick in Egypt. The molecular taxonomy of tick species is going to be a standard approach for confirming the morphological identification of ticks. Molecular detection of borrelial DNA showed a relatively low rate of *B. burgdorferi* infection in dogs and associated ticks. However, dogs act as a potential sentinel carrier of Lyme disease. Since *B. burgdorferi* is of zoonotic importance, routine monitoring of domestic animals, adequate control measures, and increased awareness of the possible Lyme Borreliosis infections are required for consideration of this pathogen in the differential diagnosis of infectious diseases.

## Methods

### Sampling

#### Ticks collection

**Fig. 4:**
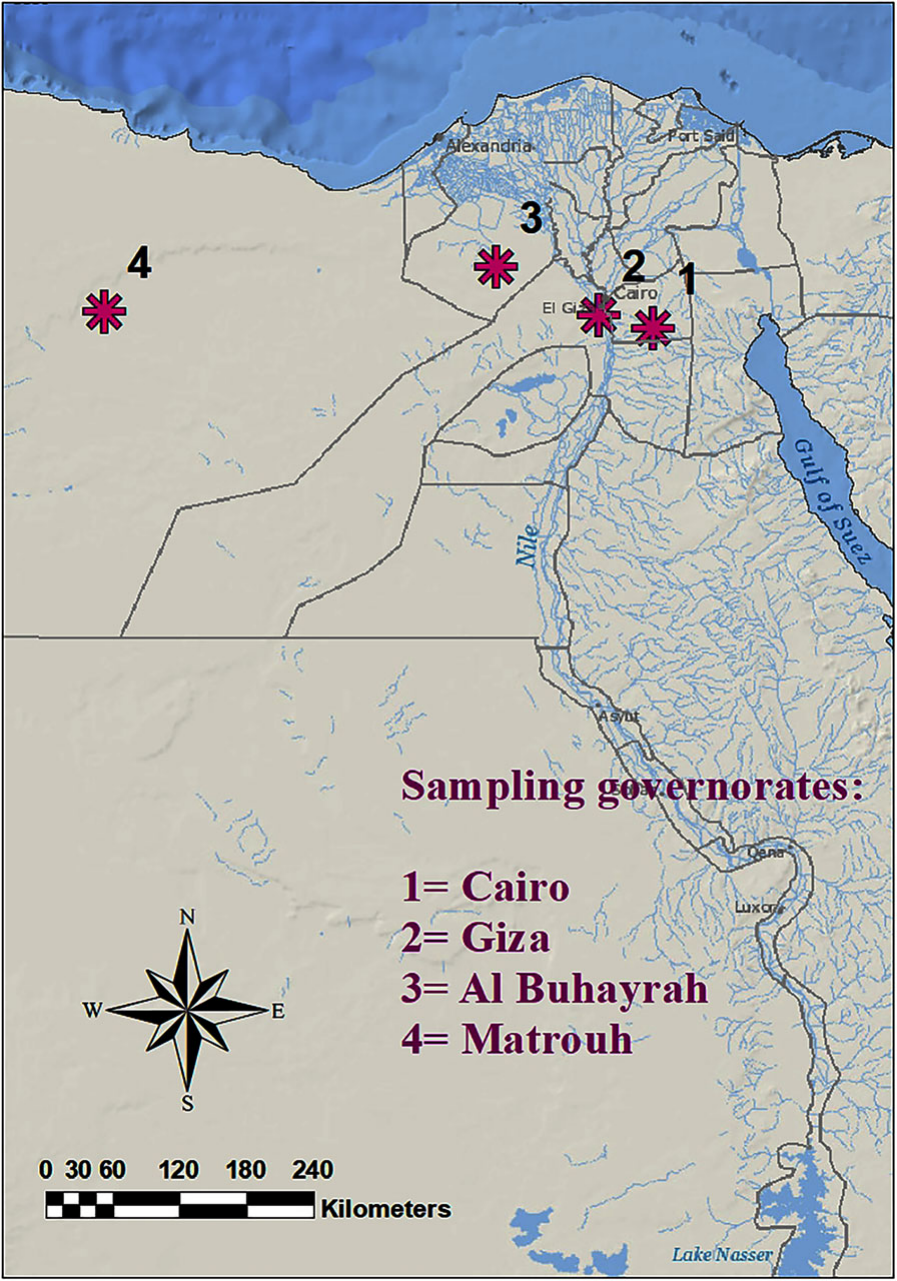
Map of sampling locations, Egypt. It was created with ArcMap version 10.1 software

A three hundred Dogs (stray and pet dogs), cattle (native breed), and camels (*Camelus dromedaries*), 100 animals each, from Cairo, Giza, Al-Buhayrah, and Matrouh governorates, were inspected for tick infestation, for 1 year (2017). Adult hard tick samples were collected and isolated from those naturally infested animals, as indicated in the (Table 3) and (Fig. 4). Ticks were pulled off manually from the animals by hands and with blunt point forceps, then placed in a sterile loosely capped plastic vials and transported to the laboratory in a dry icebox. In the laboratory, the ticks were identified and stored at − 20 °C.

**Table 3:** Blood and tick samples collected from examined animals

	Locations	No. of inspected animals for tick infestation	No. of samples		
			Blood (Infested animals)	Ticks	Tick pools
**Dog** (Stray dogs & different Breeds)	**Giza** **Cairo**	100	60	270	60
**Cattle** (Native breeds)	**Giza** **Elbehira**	100	50	390	50
**Camel** (*Camelus dromedarius*)	**Cairo** (Bassatin abattoir) **Matrouh**	100	50	365	50
**Total**		300	160	1025	160

#### Blood sampling

Blood samples were collected from hard tick-infested animals, including dogs (*n* = 60), cattle (*n* = 50), and camels (*n =* 50) from some Egyptian governorates, which are shown in (Table 3) and (Fig. 4). The blood samples were collected in tubes coated with EDTA from the cephalic vein in dogs, and the Jugular vein in cattle and camels. All blood samples were transported to the laboratory in an icebox and stored at − 20 °C until use.

### Tick identification

#### Morphological identification of ticks

Tick samples were kept at room temperature and then washed twice with sterile normal saline to remove excess particulate contamination from animal skin, rinsed once with 70% ethanol. They were mounted on slides and examined using a stereoscope microscope (BOECO, Germany). Adult ticks were identified into genera, species, and subspecies by using appropriate identification keys of morphological shapes [33]. About 20 ticks were taken from each dog and about 30–35 ticks were taken from each cattle or camel as a target number of ticks from each animal, we did not collect all the ticks infesting on each animal, which is considered a limitation of the study. The identified ticks were transferred to sterile vials and stored at − 20 °C until processing.

### Molecular identification of ticks

#### Ticks’ DNA extraction

DNA was extracted from each morphologically identified tick sample, after being crushed in a mortar with liquid nitrogen into small pieces, by using a DNA extraction kit (DNeasy Blood & Tissue Kit, QIAGEN; Germany) according to the manufacture’s protocol.

#### Tick identification by PCR

Genomic DNA extracted from the ticks’ tissues was amplified using primers designed for ITS2 amplification [34]; forward 5′-YTGCGARACTTGGTGTGAAT-3′ and reverse 5′- TATGCTTAARTTYAGSGGGT-3′ (Bio Basic Inc., Canada).

PCR conditions was done according to Muruthi et al., 2016 [35]. Amplification reactions were visualized on a 1% agarose gel containing 0.5 μg/ml ethidium bromide (Sigma), while a 1 kb DNA ladder (Invitrogen™) was used as a marker. The gel was visualized under UV light with a transilluminator.

#### Sequencing of ticks’ PCR products and phylogenetic analysis

PCR products of the identified ticks were purified from the reactions using the Qiaquick purification kit for tissues (Qiagen, Germany) according to the manufacturer’s instructions. Sequencing was conducted using Big Dye Terminator V3.1 sequencing kit (Applied Biosystems) with forward primer ITS2 ribosomal DNA. Phylogenetic analysis was based on the ITS2 region sequences of ticks. The trees were constructed and analyzed by neighbor-joining.

### Molecular detection of *Borrelia burgdorferi* 16S *rRNA* gene in blood and tick samples

#### DNA extraction from blood and ticks

DNA was extracted from blood samples using QIAamp DNA Blood Mini Kit (Qiagen, Germany), and from tick samples using QIAamp DNA Mini Kit for tissue (Qiagen, Germany) according to the manufacturer’s instructions. The DNA extract was stored at − 20 °C until being used in the PCR assay.

#### Detection of *Borrelia burgdorferi* by PCR

PCR was performed using BbF and BbR PCR primers of the *16S rRNA* gene, according to [36], in a reaction volume of 50 μl, containing 0.625 U of Taq DNA polymerase (Thermo Fischer), 2.5 mM MgCl_2_, 100 μM of deoxynucleoside triphosphates (dNTP), 12.5 pmol of species-specific primers for *B. burgdorferi* BbF 5′-GGGATGTAGCAATACATTC-3′ Position (72–90) and BbR 5′- ATATAGTTTCCAACATAGG-3′ Position (631–649), 5 μl extracted DNA from blood samples and ticks.

PCR with species-specific primers consisted of an initial denaturation (1 min at 94 °C), followed by 35 cycles using the temperature profile 95 °C for 1 min, 50 °C for 1 min and 72 °C for 1.5 min. Negative control samples (no template DNA) which were subjected to identical procedures, were used to monitor contamination, the expected molecular weight is 577 bp. 10 μL of the amplification products were analyzed on ethidium bromide-stained 1.5% agarose gel in TAE buffer (0.04 M tris-acetate, 0.002 M EDTA, pH 8) after horizontal electrophoresis at 80 V for 45 min [37, 38].

#### Sequencing of *B. burgdorferi* PCR product and phylogenetic analysis

PCR products of positive *B. burgdorferi* samples (blood and ticks) were purified from the reactions using the Qiaquick purification kit (Qiagen, Germany) according to the manufacturer’s instructions. Sequencing was conducted using Big Dye Terminator V3.1 sequencing kit (Applied Biosystems) with forward primer *16S rRNA*. The obtained nucleotide sequence was compared with those available in public domains using NCBI, BLAST server. Sequences were downloaded and imported into BioEdit version 7.0.1.4 for multiple alignments using the Clustal W program of the BioEdit. Phylogenetic analysis was performed with MEGA version 7 using the neighbor-joining method. The bootstrap consensus tree was inferred from 950 replicates (Fig. 3).

The phylogenetic tree was constructed based on the *16S rRNA* gene sequences recovered from *Borrelia burgdorferi* strains isolated from different sources, including Dog (MH685928) and Tick (MH685927) and aligned with the other related *16S rRNA* gene sequences obtained from GenBank NCBI-BLAST.

### Statistics and spatial data

Analysis of data was performed with PASW Statistics, Version 18.0 software (SPSS Inc., Chicago, IL, USA). Data collected were analyzed using both the descriptive statistic (frequencies) and the Chi-square (*χ*^*2*^) test for independence to examine the relation between animal species and tick infestation rates. A *P*-value < 0.05 was considered statistically significant. The map was created with ArcMap version 10.1 software.

The study was performed according to the guidelines of the ethical committee (Institutional Animal Care and Use Committee), Faculty of veterinary medicine, Cairo University, Vet CU. IACUC (VetCU1022019070).

## Data Availability

The datasets generated or analysed during the current study are available in the [GENE BANK] repository, accsession numbers: [MH685928, MH685927, MH685437, MH685931, MH685932].

## Abbreviations

LB: Lyme Borreliosis
Bb: *Borrelia burgdorferi*
TBD: Tick-borne disease
